# *Burkholderia pseudomallei* in Water Supplies, Southern Thailand

**DOI:** 10.3201/eid2011.140832

**Published:** 2014-11

**Authors:** Janjira Thaipadungpanit, Wirongrong Chierakul, Worawut Pattanaporkrattana, Anusorn Phoodaeng, Gumphol Wongsuvan, Viriya Huntrakun, Premjit Amornchai, Supawat Chatchen, Rungrueng Kitphati, Vanaporn Wuthiekanun, Nicholas P.J. Day, Sharon J. Peacock, Direk Limmathurotsakul

**Affiliations:** Mahidol University, Bangkok, Thailand (J. Thaipadungpanit, W. Chierakul, G. Wongsuvan, V. Huntrakun, P. Amornchai, S. Chatchen, V. Wuthiekanun, N.P.J. Day, S.J. Peacock, D. Limmathurotsakul);; Koh Phangan Hospital, Surat Thani, Thailand (W. Pattanaporkrattana, A. Phoodaeng);; Ministry of Public Health, Nonthaburi, Thailand (R. Kitphati); University of Oxford, Oxford, UK (N.P.J. Day);; University of Cambridge, Cambridge, UK (S.J. Peacock)

**Keywords:** melioidosis, B. pseudomallei, water, Phangan, Thailand, bacteria, Koh Phangan

**To the Editor:** Melioidosis is an infectious disease caused by the environmental gram-negative bacillus *Burkholderia pseudomallei*, which is present in northern Australia and across much of Asia (*1*,*2*). In Thailand, melioidosis is highly endemic to the northeast, where most infected persons are agricultural farmers with repeated environmental exposure (*3*). Melioidosis is infrequently reported from southern Thailand, although a cluster of 6 cases occurred in Phangnga Province after the December 2004 tsunami (*4*). Given the infrequency of reported cases, a cluster of 11 persons with melioidosis on Koh Phangan (an island in the Gulf of Thailand) during January–March 2012 (*5*) led to an investigation. Three case-patients were foreign tourists; 8 Thai case-patients were from 7 different villages throughout the island, and none were agricultural workers (*5*). Three cases were fatal; water inhalation was suspected as a route of infection in a fatal case in a neonate who was born in a birthing pool outside of a hospital (Technical Appendix Table 1). The lack of history for environmental exposure, such as farming, led to the hypothesis that water was the source of infection. After a request by Koh Phangan Hospital and the Thai Ministry of Public Health, an environmental survey was conducted for *B. pseudomallei* in water supplies on the island.

In March 2012, we randomly collected water from accessible water supplies in local residences and hotels from all 14 villages on Koh Phangan. A total of 190 samples were collected (range 10–18 samples per village, Figure) for culture, genotyping, and analysis (Technical Appendix). Isolates from 3 persons who died (a single bacterial colony saved from each person) from Koh Phangan were also available for genotyping and analysis. 26 (14%) of 190 samples were culture positive for *B. pseudomallei*. The positivity rate did not differ by source of the water sample: spring (5 [28%] of 18 samples), well (17 [13%] of 127), and tap water (4 [9%] of 45; p = 0.16, Fisher exact test). Of the 26 samples, 16 (62%), 9 (34%), and 1 (4%) were from local residences, hotels, and an ice cream shop, respectively. Positive water samples were distributed across the island (Figure). The median quantitative *B. pseudomallei* count was 30 CFU/L (range <10–11,300 CFU/L). The quantitative count did not differ by sample source (p = 0.16, Kruskal-Wallis test), and the sample with the highest quantitative count (11,300 CFU/L) was from well water. Of the 26 samples, only 1 was from a source that was consumed as drinking water.

**Figure:**
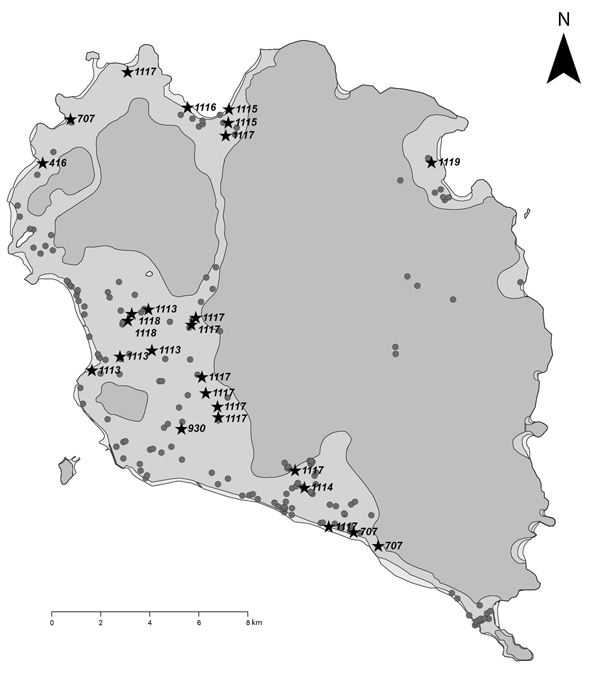
Location and multilocus sequence types of *Burkholderia pseudomallei* from water supplies on Koh Phangan, Thailand, 2012. A total of 190 water samples are indicated on the map. Twenty-six samples that were culture positive for *B. pseudomallei* are shown by black stars together with the sequence type, and 164 samples that were culture negative for *B. pseudomallei* are indicated as gray dots.

We identified 12 multilocus sequence types (STs): 10 STs from water samples and 2 different STs from 3 clinical isolates (Technical Appendix). The most frequent ST (ST1117, 10 isolates) was widely distributed across the island (Figure; Technical Appendix Table 2). Phylogenetic analysis showed 12 genetically diverse STs identified on Koh Phangan and separate clusters of the clinical and environmental isolates (Technical Appendix Figure).

Public tap water contaminated with *B. pseudomallei* has been reported previously in northeastern Thailand (*6*). The country’s National Tap Water Quality Assurance Program does not include *B. pseudomallei* (*7*), a situation that warrants review. A combination of filtration and chlorination is recommended for treatment of village tap water systems in Thailand, but recent studies report that the quality of village tap water is suboptimal (*8*). Chlorination with sufficient contact time and free available chlorine can kill *B. pseudomallei* (*9*,*10*).

We reported our findings to Koh Phangan Hospital, Koh Phangan Public Health Office, and the Thai Ministry of Public Health. Our findings led to advice being provided by Thai Ministry of Public Health to every water treatment plant, household, and hotel on Koh Phangan in April 2012 to appropriately chlorinate water before general consumption. We recommend that residents and tourists to this island drink bottled or boiled water to prevent melioidosis and other waterborne infectious diseases.

Our finding that drinking water contained *B. pseudomallei* provides evidence for ingestion as a route of infection. Other routes include skin inoculation and inhalation, but we have no evidence from the clinical history to support this, other than possible inhalation in the case of the neonate born in a birthing pool. We did not find matching genotypes in water supplies and human samplesPossible explanations include the following: the considerable genetic diversity of *B. pseudomallei* found in water in this study and elsewhere (*6*), the small sample size, and that fact that we genotyped a single colony per sample when the sample could contain multiple genotypes.

Culture-positive water samples originated from different water sources and were distributed across the island; the genotyping results were consistent with endemic infection and ruled out a single outbreak. Soil sampling and a case–control study on Koh Phangan might provide a more extensive analysis of activities associated with development of melioidosis in this setting.

Technical Appendix Water sample collection, culture, genotyping, and phylogenetic tree analysis; clinical characteristics of 3 fatal cases and sequence types (ST) of *Burkholderia pseudomallei* isolates; multilocus STs of *B. pseudomallei* from water supplies on Koh Phangan island, Thailand; and phylogenetic tree of *Burkholderia* spp.
