# Supplementary material for: Burkholderia pseudomallei in Water Supplies, Southern Thailand
Source: Emerg Infect Dis. 2014 Nov;20(11):1947–9. doi: 10.3201/eid2011.140832 (PMC4215545; doi:10.3201/eid2011.140832)
Supplement: Technical Appendix — Water sample collection, culture, genotyping, and phylogenetic tree analysis; clinical characteristics of 3 fatal cases and sequence types (ST) of Burkholderia pseudomallei isolates; multilocus STs of B. pseudomallei from water supplies on Koh Phangan island, Thailand; and phylogenetic tree of Burkholderia spp. [file 14-0832-Techapp-s1.pdf]

DOI: http://dx.doi.org/10.3201/eid2011.140832

Burkholderia pseudomallei in Water Supplies on Resort Island, Southern Thailand

Technical Appendix

Technical Appendix Table 1. Clinical characteristics of 3 fatal cases and sequence types of *Burkholderia pseudomallei* isolates

Case-patient	Age	Sex	Nationality	Occupation	Underlying disease	Sequence type
1	5 d	M	Russian	Newborn	Natural childbirth in an unknown source of water and without routine medical interventions	385
2	51 y	M	French	Tourist	Chronic liver disease	164
3	49 y	M	Thai	Labourer	None	164

Water Sample Collection, Culture, Genotyping, and Phylogenetic Tree Analysis

We collected 1.5 L water from the main water supply at each location. Householders were asked whether the water supply was used for drinking and, if so, whether it was treated before consumption. If the water supplies were treated (by filtration or boiling) before drinking, only treated water samples were collected for culture. The location of each water sample was recorded by using EpiCollect (*1*), and a map was drawn using ArcMap implemented with ArcGIS 10.0 for desktop (ESRI, Redlands, CA, USA).

For each water sample, 1, 10, and 100 mL were each passed through a 0.45-µm filter to obtain a quantitative bacterial count. The remaining 1.4 L was passed through 2.5 g of sterile diatomaceous earth (Celite; World Minerals Corporation, San Jose, CA, USA) to obtain a sensitive, qualitative method (*2*). Filters were cultured on Ashdown agar, and diatomaceous earth was cultured in selective broth (15 mL of threonine-basal salt plus colistin broth) (*2*). Broth was incubated at 40°C in air for 48 h, after which 10 µL of the upper layer was streaked onto an Ashdown agar to achieve single colonies. All Ashdown agar plates were incubated at 40°C in air, and examined every 24 h for 7 d. If enrichment broth cultures were positive for *Burkholderia pseudomallei* but filter cultures were negative, the quantitative count was defined as <10 CFU/L.

A single colony of *B. pseudomallei* isolated from each water sample was selected for genotyping by using multilocus sequence typing (MLST). MLST was undertaken as described previously (*3*). A maximum-likelihood tree was reconstructed from concatenated

sequences of 7 MLST loci by using PhyML version 3.0.1 (*4*). The CLC Main Work Blench version 7.0 was used to edit and display the tree (QIAGEN, Valencia, CA, USA).

We compared genotypes of *B. pseudomallei* from 26 water specimens and 3 melioidosis cases from Koh Phangan. Sequence types (STs) of the clinical isolates (ST385 [n = 1] and ST164 [n = 2]) have been identified previously from water in southern Thailand (ST385) (*5*), and soil in northeastern Thailand (ST164) (*6*). Three of 10 STs from water samples have been identified previously from water in southern Thailand (ST416), and human case-patients from Cambodia (ST930) and Nigeria (ST707). The remaining 7 STs were novel (ST1113 to ST1119). A total of 12 STs were identified, including 10 STs from water samples and 2 different STs from 3 clinical isolates.

A maximum-likelihood tree of *B. pseudomallei* isolates from Koh Phangan was constructed, together with all of the unique STs in the public MLST database (http://bpseudomallei.mlst.net/) (Technical Appendix Figure 1). The tree showed that the 12 STs identified on Koh Phangan were genetically diverse and fell into 6 clusters. The major cluster included 19 isolates from 5 novel STs (ST1113, ST1115–ST1118), suggesting that these related genotypes dominate on this island. The clinical and environmental isolates did not cluster together.

References

1. Aanensen DM, Huntley DM, Feil EJ, al-Own F, Spratt BG. EpiCollect: linking smartphones to web applications for epidemiology, ecology and community data collection. PLoS ONE. 2009;4:e6968. PubMed http://dx.doi.org/10.1371/journal.pone.0006968

2. Limmathurotsakul D, Wongsuvan G, Aanensen D, Ngamwilai S, Saiprom N, Rongkard P, et al. Melioidosis caused by *Burkholderia pseudomallei* in drinking water, Thailand, 2012. Emerg Infect Dis. 2014;20:265–8. PubMed http://dx.doi.org/10.3201/eid2002.121891

3. Godoy D, Randle G, Simpson AJ, Aanensen DM, Pitt TL, Kinoshita R, et al. Multilocus sequence typing and evolutionary relationships among the causative agents of melioidosis and glanders, *Burkholderia pseudomallei* and *Burkholderia mallei*. J Clin Microbiol. 2003;41:2068–79. PubMed http://dx.doi.org/10.1128/JCM.41.5.2068-2079.2003

4. Guindon S, Gascuel O. A simple, fast, and accurate algorithm to estimate large phylogenies by maximum likelihood. Syst Biol. 2003;52:696–704. PubMed http://dx.doi.org/10.1080/10635150390235520

5. McCombie RL, Finkelstein RA, Woods DE. Multilocus sequence typing of historical Burkholderia pseudomallei isolates collected in Southeast Asia from 1964 to 1967 provides insight into the epidemiology of melioidosis. J Clin Microbiol. 2006;44:2951–62. PubMed http://dx.doi.org/10.1128/JCM.00725-06

6. Vesaratchavest M, Tumapa S, Day NP, Wuthiekanun V, Chierakul W, Holden MT, et al. Nonrandom distribution of *Burkholderia pseudomallei* clones in relation to geographical location and virulence. J Clin Microbiol. 2006;44:2553–7. PubMed http://dx.doi.org/10.1128/JCM.00629-06

Technical Appendix Table 2. Multilocus sequence types of *Burkholderia pseudomallei* from water supplies on Koh Phangan, Thailand, 2012

Sequence	No.			
type	Tap water	Well water	Spring water	Total
416	0	1	0	1
707	0	1	2	3
930	0	1	0	1
1113	0	4	0	4
1114	0	1	0	1
1115	1	0	1	2
1116	0	1	0	1
1118	0	2	0	2
1119	0	0	1	1
1117	0	3	6	10
Total	4	17	5	26

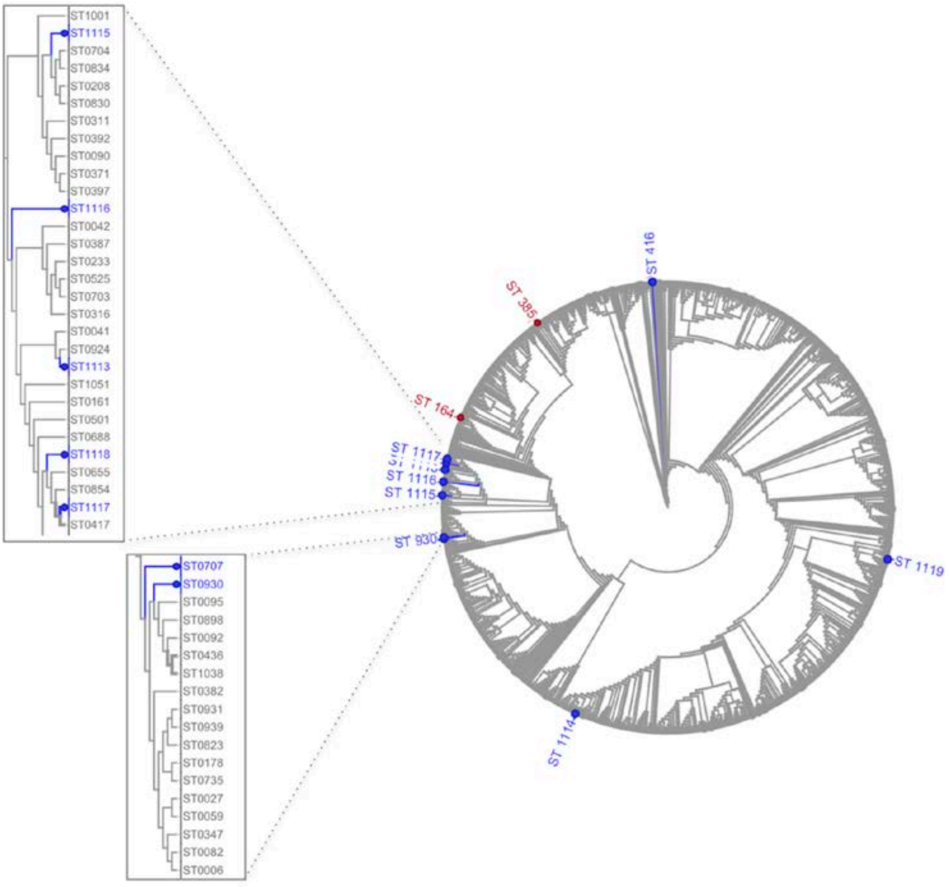

Technical Appendix Figure. Phylogenetic tree of *Burkholderia* spp. A maximum likelihood tree was reconstructed from concatenated nucleotide sequences of 7 multilocus sequence type loci. The isolates included were *B. pseudomallei* from Koh Phangan island, Thailand, as described in this study, together with 1 representative of each of the sequence types (STs) held in the public *Burkholderia* MLST database (http://bpseudomallei.mlst.net/). Blue dots indicate STs found in water supplies; red dots indicate STs isolated from the 3 patients with fatal melioidosis.
